# Perchloride of Iron as a Hæmostotic

**Published:** 1865-02

**Authors:** 


*Perchloride of Iron as a Hæmostotic.*—The *Antwerp Journal* states that perchloride of iron combined with collodion is a good hæmostatic in the case of wounds, the bite of leeches, &c. To prepare it, one part of crystalized perchloride of iron is mixed with six parts of collodion. The perchloride of iron should be added gradually and with care, otherwise such a quantity of heat will be generated as to cause the collodion to boil. The composition when well made is of a yellowish-red color, perfectly limpid, and produces on the skin a yellow pellicle, which retains great elasticity.

